# Adjuvant Chemotherapy Does Not Compensate for an Inadequate Right Colon Cancer Surgery: High Peritoneal Recurrence Rates Indicate Need for Altered Treatment Paradigms

**DOI:** 10.1007/s13193-024-02099-2

**Published:** 2024-09-25

**Authors:** Swapnil Patel, Mufaddal Kazi, Anand Mohan, Vivek Sukumar, Ashwin L. deSouza, Avanish Saklani

**Affiliations:** 1 Department of Surgical Oncology, MPMMCC & HBCH, Tata Memorial Centre, Varanasi, India; 2 Colorectal Division, Department of GI & HPB Surgery, Tata Memorial Centre, Homi Bhabha National University, Mumbai, 400012 India; 3 Department of Surgical Oncology, Mahatma Gandhi Hospital, Jaipur, India; 4 Department of Surgical Oncology, Upkar Cancer Institute, Varanasi, India

**Keywords:** D3 lymphadenectomy, Complete mesocolic excision, Central vascular ligation, Right colon cancer

## Abstract

There is a lack of evidence for optimal management of patients with right colon cancers upon referral to the oncology care centre, following an inadequate index surgery elsewhere. A prospectively maintained database of patients with right colon cancers managed between 2013 and 2019 was screened to identify those patients who underwent index surgery in a non-oncological setup. They were managed with adjuvant chemotherapy followed by observation, with surgery being reserved for recurrent disease. Of the 155 patients identified after the screening, 97 were included in the study. They were stratified depending upon the number of lymph nodes harvested at primary surgery—Group A (less than 12 nodes) (*n* = 49), Group B (12 to 27 nodes) (*n* = 39) and Group C (28 and more nodes) (*n* = 9). Patients with lymph node metastases had inferior survival at 2 years than node-negative patients and this survival difference increased progressively from Group A towards Group C. Patients who had radiological locoregional residual disease upon restaging (at presentation) had significantly inferior survival. At the end of 2 years, overall survival and disease-free survival of the cohort were 71.5% and 45.8%, respectively. Fifty-eight patients had disease relapse, with peritoneal recurrence seen in 37 patients (63.8%). Of these, only 15.5% recurrences were surgically salvageable. Treatment of patients who have undergone inadequate index colectomy with chemotherapy alone has shown inferior survival outcomes with high rates of peritoneal relapse in comparison to historical cohorts. The treatment strategy for such patients needs to be revisited in a prospective study design.

## Introduction

Complete mesocolic excision (CME) and central vascular ligation (CVL) have been suggested as the standard surgery for the management of right colon cancers by Hohenberger [1]. Following similar principles, D3 lymphadenectomy has been propagated by Japanese surgeons [2]. These technical advancements have translated into lower local recurrence rates with superior overall survival (OS) [3]. The ongoing debate around the radicality of lymph node dissection (D2 versus D3 lymphadenectomy) seems to be settling in favour of CME surgery [4–6]. However, due to a lack of availability of technical expertise, patients often undergo non-CME surgery in community settings. This holds true, especially in those countries where referral pathways from the community are not defined. Upon referral to specialized centres, patients with index non-CME colectomy are usually offered chemotherapy, without any assessment for subsequent re-look or revision surgery. There is no available report on the outcomes of such patients. The current study has analyzed a cohort of patients who underwent index non-CME colectomy in the community setting and then subsequent treatment with chemotherapy alone at a specialized centre.

## Methods

This is a retrospective study, conducted at a single tertiary cancer care centre. Patients with right colon cancer, who underwent index inadequate colectomy (as subsequently defined by authors) and then treated at the author’s institute between 2013 and 2019, were included in the study.

### Inclusion Criteria

1. Patients who underwent index surgery in a community setting (non-oncological setup)
2. Patients with right colonic adenocarcinoma
3. Patients who completed at least 6 months of follow-up after completion of planned treatment

### Exclusion Criteria

1. Patients who had distant metastases documented at index surgery
2. Patients who presented at the author’s institute with a poor performance status (ECOG 3 and above)
3. Patients who were operated on outside under labelled emergency conditions (obstruction or bleeding)

### Primary Objective

1.1. To analyze the survival estimates of the cohort—overall survival (OS) and disease-free survival (DFS).

### Secondary Objective

1. To study the patterns of recurrence and the salvage rates of recurrent disease
2. To analyze the impact of lymph node dissection (as a surrogate of completeness of CME) on survival

### Disease Management Protocol

Patients who underwent index surgery elsewhere were restaged upon referral, with a contrast-enhanced computed tomography scan of the chest, abdomen and pelvis. The further treatment was decided based on the adequacy of the index surgery, the presence of residual disease (locoregional disease and or distant metastatic disease) upon restaging and the performance status of the patient. Histopathological details of the tumour from the index surgery were noted and reviewed by the pathologists at the authors’ institute. Adequacy of the surgery was defined based on free resection margins (5- to 10-cm longitudinal tumour-free margin and at least 1-mm circumferential resection margin), lymph node harvest (minimum 12 lymph nodes dissected) and an R-0 resection status at the index surgery.

Patients with adequately staged early disease were kept on observation and followed up at regular intervals. Patients with any regional lymph node metastases or those with inadequate lymph node harvest (less than 12 lymph nodes) were offered adjuvant chemotherapy (6-month course of modified FOLFOX6 regimen or Cape-Ox regimen) [7]. Patients with resectable locoregional disease or distant metastases upon restaging were planned for surgery after chemotherapy [8]. Patients with unresectable locoregional or distant metastatic disease were offered chemotherapy with palliative intent.

### Statistical Analysis

Statistical analyses were performed using SPSS Version 25. The baseline demographic and treatment characteristics were recorded. The distribution of the characteristics was seen between two groups of patients—adequate lymph node dissection (less than 12 nodes) and inadequate lymph node dissection (12 or more nodes) as per the AJCC 8th edition. Student *t*-test was used for comparison of nominal data while the chi-square test was used for comparing continuous variables and proportions.

For a sub-group analysis, the cohort was subdivided into three categories depending upon the number of lymph nodes harvested at primary surgery—Group A (less than 12 nodes), Group B (12 to 27 nodes) and Group C (28 or more nodes). This sub-group analysis was done to analyze the impact of lymph nodes dissected on survival outcomes (as per Hohenberger’s criteria) [1].

Overall survival (OS) was calculated from the date of index surgery to death due to any cause. Disease-free survival (DFS) was calculated from the date of index surgery to the first evidence of recurrence. Kaplan Meier curves were used for survival analyses.

The manuscript has been reviewed by the Institutional Ethics Committee and has been performed in accordance with the ethical standards laid down in the Declaration of Helsinki (as revised in Brazil 2013).

## Results

### Cohort Description

**Fig. 1:**
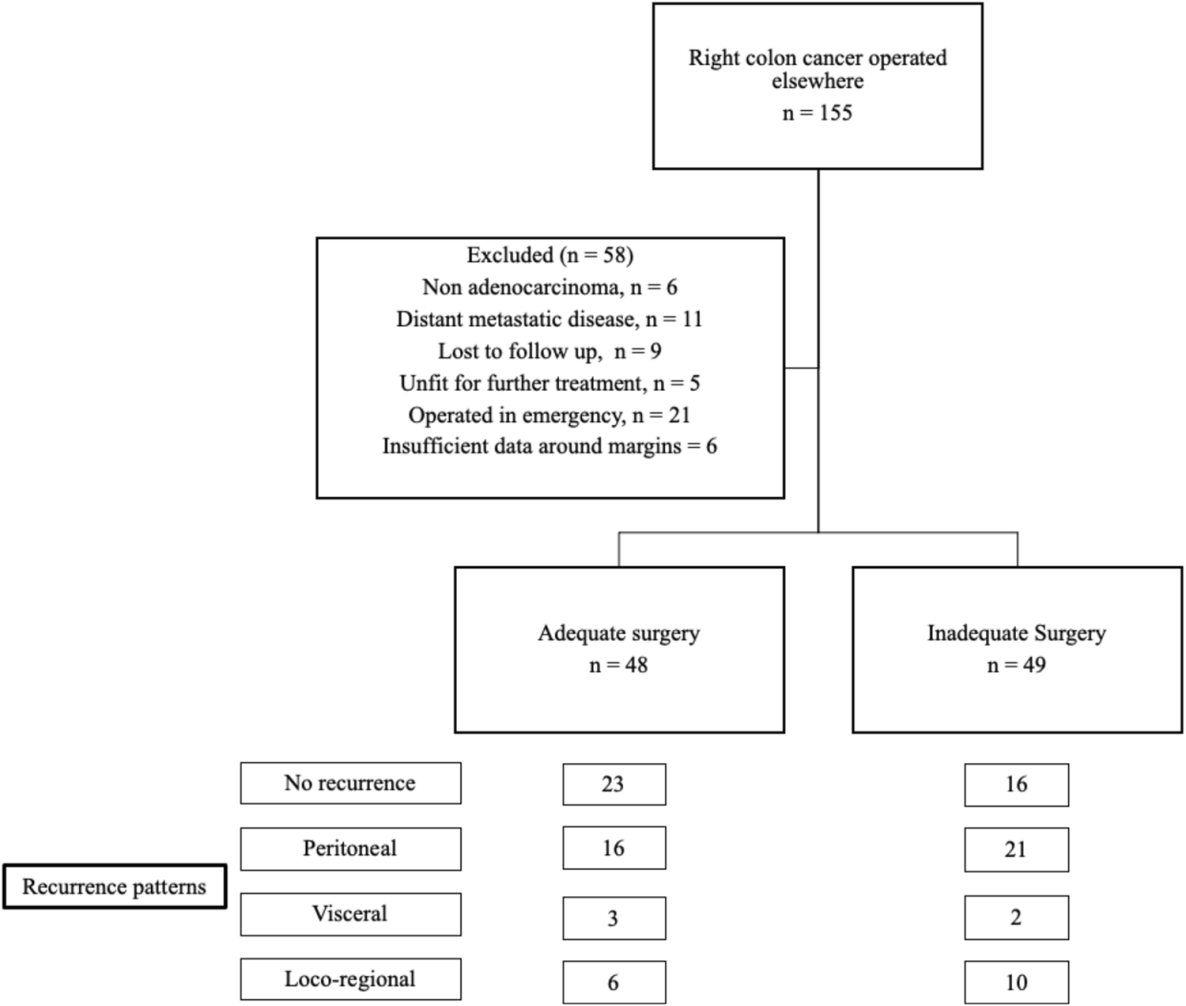
Cohort diagram

A total of 155 patients who were referred to the institute after surgery elsewhere were identified. Of these, 97 patients were found eligible for the study as per the aforementioned criteria (Fig. 1).

The median age was 51 years (11 to 76 years) and the male:female ratio was 2:1. The histopathology data from the initial surgery was collected and analyzed. The median lymph nodal harvest of the overall cohort was 11 nodes. With respect to baseline nodal staging after surgery, 49 patients did not have the required information and were labelled as Nx (inadequate lymph node dissection group). Amongst the remaining 48 patients, the nodal staging was as follows: N0–17, N1–16, N2–15. This group was labelled as the adequate lymph nodal dissection group. The distribution of baseline characteristics amongst the two groups is shown in Table 1. The treatment characteristics (other than nodal staging) were equitably distributed across both groups. The longitudinal margins were reported free for all the patients included in the cohort. All the surgeries were performed using an open approach, in an elective setting.

**Table 1:** Comparison of baseline characteristics and treatment characteristics between patients with inadequate lymph node dissection (LND) and adequate LND

	Inadequate LND	Adequate LND	*p* value
Number of patients	49 (50.5%)	48 (49.5%)	
Age < 50 years > 50 years	21 (21.6%) 28 (28.9%)	26 (26.8%) 22 (22.7%)	0.265
Sex Male Female	31 (32.0%) 18 (18.6%)	34 (35.1%) 14 (14.4%)	0.428
Grade* WD MD PD Signet ring cell	5 (5.2%) 27(27.8%) 9 (9.3%) 8 (8.2%)	3 (3.1%) 36 (37.1%) 4 (4.1%) 5 (5.2%)	0.222
pT stage pT1 pT2 pT3 pT4	1 (1.0%) 5 (5.2%) 29 (29.9%) 14 (14.4%)	0 (0%) 3 (3.1%) 27 (27.8%) 18 (18.6%)	0.608
Local residual disease upon restaging	5 (5.2%)	5 (5.2%)	0.869
Delay in presentation < 6 weeks > 6 weeks	37 (38.1%) 12 (12.4%)	34 (35.1%) 14 (14.4%)	0.603

^*^WD well differentiated, *M*D moderately differentiated, *PD* poorly differentiated

### Survival Analysis

**Fig. 2:**
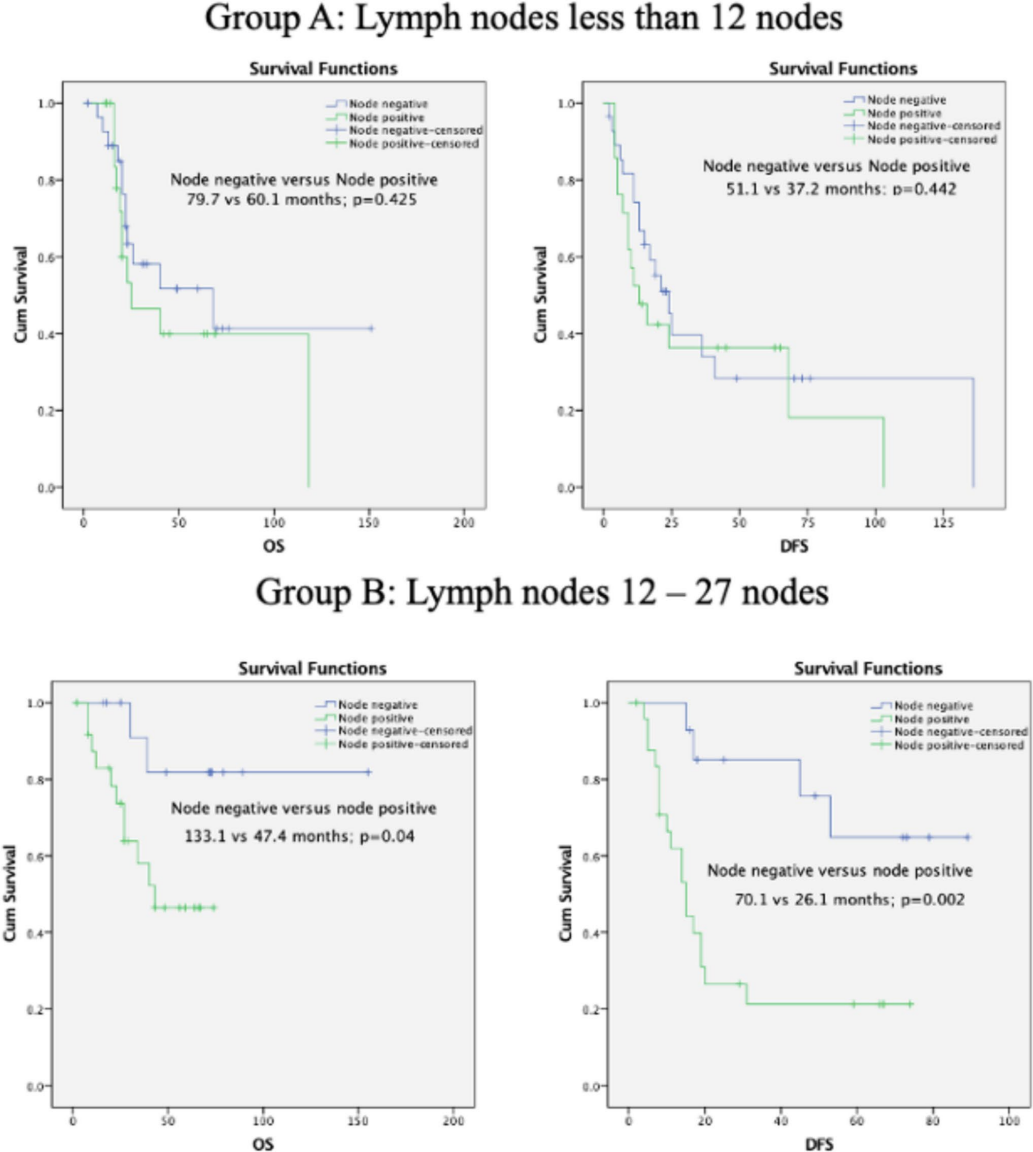
Survival graph and Kaplan–Meier estimates of patients with lymph node metastases compared to patients with no lymph node metastases segregated for the numbers of lymph nodes dissected (**A**) less than or equal to 11 nodes dissected—DFS (B) OS (C) 12 to 27 nodes dissected—DFS (D) OS (E) 28 or more nodes dissected—DFS (corresponding OS curve not applicable)

At the end of 2 years, the OS of the overall cohort was 71.5% and the corresponding DFS was 45.8%. The survival of the cohort was further analyzed with respect to the involvement of lymph nodes by metastatic disease amongst the three groups A (*n* = 49), B (*n* = 39) and C (*n* = 9). Amongst patients in Group A, there was no statistical difference in survival (OS and DFS) between patients with negative and positive lymph nodes. In Group B, patients who had lymph node involvement had inferior survival OS (*p* = 0.04) and DFS (*p* = 0.002) as compared to patients who did not have any lymph node involvement. In Group C, the survival difference was further increased in favour of patients with no lymph node involvement. There were no deaths in either of the groups, and there were no recurrences in lymph node–negative patients; hence, corresponding survival estimates are not available (Fig. 2).

Survival analyses were also carried out amongst the sub-group of patients with and without residual disease upon restaging. Patients who had residual disease post-index surgery fared worse. The OS and DFS were significantly inferior to the patients with no residual disease. Patients with the residual disease had a median OS of 20 months in comparison to a median OS of 118 months for patients with no residual disease post-index colectomy (*p* value = 0.000). Similarly, the patients with residual disease had significantly inferior DFS (8 versus 26 months; *p* = 0.004).

### Recurrence Patterns

Patients who had recurrences (*n* = 58) during the study period were analyzed. The patients failed at various sites in the following order: peritoneal (*n* = 37, 63.8%), locoregional (*n* = 16, 27.6%) and visceral (*n* = 5, 8.6%).

The treatment offered for recurrence was analyzed with respect to the subsite of recurrence (Table 2) (*p* = 0.000). Overall, 15.5% recurrences were salvageable. Only 7 out of 16 patients (43.7%) with locoregional recurrence could be offered salvage surgery. Many patients (12 out of 37, 32.4%) with peritoneal recurrences were not fit for any treatment at the time of recurrence and received only supportive treatment.

**Table 2:** Sites of relapse with the corresponding treatment for the analyzed cohort

Site of relapse	Recurrence treatment				Total
	Chemotherapy	Surgery	CRS + / − HIPEC	Best supportive care	
Peritoneal	18	0	7	12	37
	31.0%	0.0%	12.1%	20.7%	63.8%
Visceral	3	2	0	0	5
	5.2%	3.4%	0.0%	0.0%	8.6%
Locoregional	8	7	0	1	16
	13.8%	12.1%	0.0%	1.7%	27.6%
Total	29	9	7	13	58
	50.0%	15.5%	12.1%	22.4%	100.0%

*CRS* cytoreductive surgery, *HIPEC* hyperthermic intra-peritoneal chemotherapy

## Discussion

The current study analyzed the outcomes of patients undergoing inadequate surgery in a non-oncological setup and then subsequently managed with chemotherapy alone. At the end of 2 years, the OS of the cohort was 71.5% and the corresponding DFS was 45.8%. An alarming 58 out of 97 patients (59.8%) suffered early disease relapse (first 2 years), with peritoneal relapse being most common in 37 patients (38.1%). When patients had inadequate nodal harvest (less than 12 nodes), survival was similar between patients with lymph node metastases and negative lymph nodes. With the increasing harvest of lymph nodes, the group with lymph node metastases had progressively decreased survival in comparison to those with negative lymph nodes.

Hohenberger et al. introduced the concept of CME for right colon cancers, citing the number of lymph nodes dissected to be a surrogate of surgical adequacy [1]. They suggested retrieval of 28 lymph nodes as an adequate nodal harvest in right colon cancer. Upon dissection of more than 28 nodes, patients had significantly superior survival outcomes, irrespective of the involvement of nodes with metastatic disease. The concepts of CME with CVL and D3 lymphadenectomy propagated by the West and East groups of surgeons, respectively, aim at a higher nodal yield after radical surgery. In principle, the concept of CME and D3 lymphadenectomy proposes thorough clearance of the lympho-vascular packet while removing the central nodes. In the current study, authors hypothesized that inadequate clearance should lead to higher chances of peritoneal metastases secondary to tumour spillage caused by disruption of lymphatic channels, especially in those having metastatic regional lymph nodes. With this hypothesis in mind, this study is an attempt to shed light on the outcomes of patients who underwent inadequate index surgery in the community setting.

In a recent publication from the same institute, authors have elucidated the outcomes of patients with right colon cancers treated with CME and CVL at the institute [9]. Desouza et al. have shown that patients undergoing adequate right colon surgery have a 5-year OS and DFS of 87.5% and 80.4%, respectively. Thirty-seven of 244 (15.2%) patients suffered relapse at a median follow-up of 62 months, with peritoneal relapse being seen in 6.1% patients. Distant visceral metastases were the most common site of relapse (7%). Similarly, in other published literature, locoregional and peritoneal recurrences are usually seen in 4% and 4.5% cases, respectively [10–12]. However, the current study reports peritoneal recurrences in 38.1% cases. This high rate of peritoneal relapse is alarming and probably has its basis in inadequate index surgery. Authors hypothesize that disruption of lymphatic channels during the sub-optimal surgery coupled with inadequate nodal clearance leads to an increased risk of subsequent peritoneal relapse.

This high rate of peritoneal recurrence points to an unrealized need for treatment intensification upon referral to a tertiary centre. Currently, the treatment options available for such patients presenting after an initial sub-optimal surgery include either a revision completion surgery or chemotherapy. Although there are no published reports on the management of the same, patients are usually offered chemotherapy. The main reason for deferring a revision surgery is a technically challenging dissection around the mesenteric-portal axis in an already operated surgical field.

None of the published studies have analyzed the actuarial benefit of chemotherapy in patients with incomplete primary surgery. It is unclear if chemotherapy alone could compensate for a sub-optimal surgery. Authors suggest that the practice of chemotherapy after an incomplete surgery seems more convenience-based, rather than evidence-based.

Surgery with R-0 resection offers the only chance at long-term cure of locally recurrent colon cancers [13, 14]. While such operative strategies have been shown to improve outcomes in recurrent settings, similar treatment schema may lead to improved outcomes in patients after an index incomplete surgery by removing microscopic or macroscopic residual disease. This may especially hold true for the subset of patients who are found to have radiological residual disease upon restaging at presentation. In the current study, a sub-group of patients with residual disease were found to have significantly inferior OS and DFS as compared to patients with no residual disease. Authors postulate that delaying revision completion surgery in patients with radiological residual disease with chemotherapy first approach results in increased rates of unsalvageable peritoneal metastases. In this study, only 15.5% recurrences were surgically salvageable.

Treatment and referral pathways for colorectal cancers (CRCs) have been standardized in the West [15–17]. However, a similar standardization is lacking in low-middle-income countries (LMICs). This leads to an increased burden of inadequately treated colon cancers in LMICs. While the debate continues on the aggressiveness of lymphadenectomy in per-primum cases, operative strategies need to be defined and standardized for sub-optimally treated right colon cancers. This definition of an alternative treatment strategy for inadequately treated colon cancer is the need of the hour, especially in those countries where referral patterns are not standardized.

The current study shows that the OS of the overall cohort was 71.5%, and the corresponding DFS was 45.8% at the end of 2 years. This is very low compared to the reported outcomes after adequate treatment of colon cancers [18]. In such cases, when the reported outcomes are inferior, especially in patients with residual disease after primary surgery, the option of alternative treatment (revision surgery) should be discussed with the patients.

**Fig. 3:**
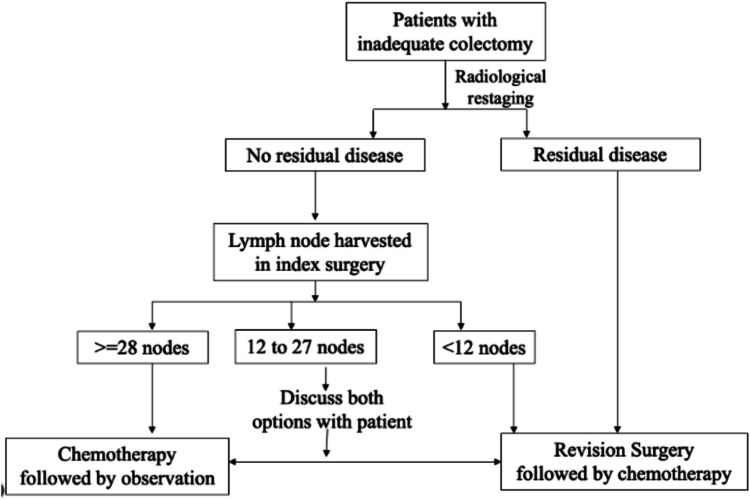
Suggested treatment algorithm for patients with an inadequate index colectomy

In this study, amongst patients with less than 12 lymph nodes harvested, patients with no lymph node involvement had similar survival as patients with involved lymph nodes. Amongst patients with 12 to 27 nodes dissected, patients with no involved nodes had significantly superior OS and DFS. Amongst patients with more than 28 nodes dissected, there were no events during the study period with significantly increased OS and DFS. Authors propose a role for revision surgery at presentation in patients with less than 12 nodes dissected at index surgery. Patients with more than 28 nodes dissected may be safely placed on routine follow-up after completion of chemotherapy. In the intermediate group (12 to 27 nodes), it may be wise to have a discussion with the patient for revision surgery versus observation after completion of chemotherapy (Fig. 3). Segregation of patients based on lymph node dissection and residual disease into varying treatment strategies (revision surgery or only chemotherapy) may provide a plausible solution to improve the disease outcomes.

This study provides the first description of outcomes of colon cancers with an index inadequate colectomy, done in non-oncological setups in inexperienced hands. The authors acknowledge poor outcomes after such inadequate preliminary surgery. It is a hypothesis-generating study which lays emphasis on the role of revision surgery as a possible strategy to improve outcomes of sub-optimally treated colon cancers. Limitations of this study include its retrospective nature along with a limited sample size. The possibility of non-documented peritoneal disease at the preliminary exploration is noted and it may be a part of recall bias inherent to the retrospective study design.

Currently, there is a lack of evidence to support the role of revision surgery in decreasing the rate of peritoneal metastases. Further trials comparing the role of adjuvant chemotherapy alone versus upfront revision completion surgery for such patients would provide the appropriate solution to the problem at hand. This study may serve as a basis for the calculation of appropriate sample size for such trials in the future. Specialized surgical units dealing with recurrent right colon cancers need to standardize the often technically challenging dissection along the mesenteric-portal axis while avoiding serious morbidity. The addition of intra-peritoneal chemotherapy during relook surgery to these patients being explored after an inadequate colectomy may be another potential area of research.

## Conclusion

Patients with right colon cancers undergoing inadequate index colectomy and then managed with chemotherapy alone have inferior survival outcomes. A higher proportion of such patients suffer a peritoneal relapse and these recurrences are often non-salvageable. The treatment strategy for patients presenting after an initial incomplete surgery needs to be revisited.

## Data Availability

The data that support the findings of this study are available upon request from the team of authors.
